# Measuring bioelectric impedance outputs following coffee consumption in healthy adults using an 8-electrode segmental BIA device

**DOI:** 10.1080/15502783.2025.2528531

**Published:** 2025-07-03

**Authors:** Christopher Chamberlin, Aldo Lena, Dimple Radia, Dale Rees, John Lodge, James Rutherford, Bruno Cesar da Silva dos Santos, Bhaven Patel, Shawn McLaren

**Affiliations:** a School of Human Sciences, London Metropolitan University, London, UK; b School of Biosciences, University of Surrey, Guildford, UK; c School of Allied Health Professionals, University of Winchester, Winchester, UK

**Keywords:** BIA, bioimpedance analysis, reliability, nutrition assessment, coffee

## Abstract

**Introduction:**

Bioelectric impedance analysis (BIA) is increasingly used to measure body composition in athletic, clinical and research settings. The reliability of BIA measurements relies on following procedures carefully. However, some practices for ensuring reliable measures may be unnecessarily restrictive. Previous research using BIA outputs as study outcome measures, has required participants to avoid coffee and caffeine-containing foods and beverages prior to measurements. The aim of this study was to determine whether BIA outputs are affected by coffee consumption at different caffeine concentrations.

**Methods:**

This study used a blinded, randomized, cross-over trial design. Participants (*n* = 13) received one of three treatments per visit: 200 mL hot water (80°C) mixed with 5 g instant coffee, 2.5 g instant coffee with 2.5 g decaffeinated coffee, or 5 g decaffeinated instant coffee. Body composition and fluid parameters were measured over 50 minutes using a Seca mBCA 515 device.

**Results:**

The treatment predictor (*p* > 0.05) and sex-time-treatment interaction for all outcomes was found to be non-significant (*p* > 0.05). The time predictor was statistically significant (*p* < 0.05) for impedance, resistance and reactance but not for phase angle ϕ50 (*p* = 0.731), ϕ5 (*p* = 0.059) or urine osmolality (*p* = 0.066). The sex predictor was statistically significant for Z_50_ (*p* = 0.001), Z_5_ (*p* = 0.002), R_50_ (*p* = 0.001), R_5_ (*p* = 0.002), ϕ_50_ (*p* = 0.01), ϕ_5_ (*p* = 0.049), fat mass (%) (*p* = 0.016) and fat free mass (%) (*p* = 0.016). The effect size for this predictor was η^2^_G_ < 0.336. A significant sex-time interaction was found for Z_50_ (*p* = 0.025) with a small effect size (η^2^_G_ < 0.01). Effect sizes for the treatment predictor and time-treatment interaction were found to be small (η^2^_G_ < 0.01). Effect size for the time predictor was small (η^2^_G_ > 0.01).

**Conclusion:**

Changes in impedance, resistance and reactance were detected over the course of the experiment, and these changes were greater than could be explained by the technical error of measurement. However, the amount of caffeine in coffee did not affect BIA outputs. Effect sizes were small, suggesting little practical significance of drinking coffee before taking BIA measurements. Therefore, coaches, athletes, researchers and clinicians may be able to obtain reliable BIA measurements even when coffee has been consumed up to 50 minutes prior to measurements, however, fluid consumption and being adequately hydrated should still be considered.

## 1. Introduction

Body composition analysis has an important role in health research. The global prevalence of overweight and obesity with associated chronic diseases is increasing [1]. Insights into body composition are valuable in understanding the interactions between fat mass fat free-mass and human health outcomes [2]. Body fat percentage is capable of predicting features of chronic diseases including hypertension, elevated LDL cholesterol, and low HDL cholesterol [3]. Body composition is an important determinant of athletic performance, and research aimed at improving athletic performance relies on an accurate understanding of fat mass and fat-free mass [4].

Bioelectric impedance analysis (BIA) is a frequently used technique for measuring body composition in clinical and research settings. This technique relies on differences in electrical impedance within a two-compartment human body model. Keys and Brozek [5] established the consistency in the density of human adipose tissue regardless of its source. A two-compartment model of body composition is postulated to comprise fat mass (FM), and fat-free mass (FFM). Therefore, estimating fat mass and fat-free mass is made possible as fat free mass has a constant water content of approximately 73% and contains electrolytes including potassium at a concentration of 50–60 mmol/kg in females and 60–70 mmol/kg in males, while fat mass is anhydrous and potassium free. Fat mass has a density of 0.9 g/mL, fat free mass density of 1.1 g/mL, assuming constant hydration, and a constant proportion of fat free mass to bone or mineral content. Fat mass and fat free mass have distinct water and electrolyte contents [6], which enables BIA as a method for measuring body composition. As FFM contains all of the body water in this model, FFM can be predicted as FFM= $\frac{\mathrm{Total}\;\mathrm{body}\;\mathrm{water}}{0.73}$. BIA works by transmitting an electric current through the body between two electrodes and measuring the difference in voltage encountered at the terminal electrode. Resistance (R; *R* = voltage (E)/current (I)), is the opposition to the flow of a current and corresponds to how well an object can transmit a current. Reactance (X) is caused by capacitance (X_c_) or inductance (X_L_), and results in the current becoming out of phase with the electrical force from which it originates [7]. In complex biological objects like the human body, the viscosity and, therefore, conductance of physiological tissues influence resistance, and reactance is affected by the presence of capacitors, including cell membranes [8]. Impedance (Z), calculated as $Z=\sqrt{R^{2}+{\left(X_{L}-X_{C}\right)}^{2}}$ is used by BIA devices to estimate body composition. Fat-free mass, made up of the aqueous tissues in the body, is a good conductor of electrical currents, whereas a fat mass that lacks water and electrolytes is a poor conductor of current. Therefore, individuals who have a high proportion of body fat will have a higher resistance to current, while individuals with a high proportion of FFM will have a lower resistance, and this is the basis of BIA. BIA devices are able to predict FFM, total body water (TBW), intracellular fluid (ICF) and extracellular fluid (ECF) using regression models that make use of resistance and reactance to calculate impedance. However, BIA makes some important assumptions about the shape and size of the human body to predict these outcomes. Impedance is related to the specific conductivity (p), length (L) and cross-sectional area (A) of the body conducting the current (Z = p(L/A)), which assumes a constant cross-sectional area, resulting in a consistent cylindrical shape [9]. This could be expressed in terms of body volume (BV) yielding BV = pL^2^/Z. Reactance has very little impact on impedance in the human body, and so it may be assumed that impedance and resistance are roughly equivalent. The relationship between FFM and TBW, established previously, shows the relationship between conductivity and body size. A larger body, with a higher FFM (including muscle and bone) has a lower resistance when corrected for height (expressed as Height^2^). Therefore, TBW can be predicted using the equation TBW = p ×Height^2^/Resistance. Different tissues have different specific conductivities, ranging from 0.7–0.8 siemens/meter (S/m) and 0.3–0.5 S/m in blood and muscle, to 0.02–0.05 S/m in fat and 0.02–0.04 S/m in bone. The human body is not a consistent, cylindrical shape, but is made up of five imperfect cylinders (the four limbs and the trunk+head), and these different cylinders have different proportions of different tissues which have different specific conductivities. These assumptions do not apply to segmental BIA devices such as the SECA mBCA 515, and Bosy-Westphal et al. [10] have demonstrated a high degree of accuracy in these devices compared with traditional wrist-to-ankle devices.

The accuracy and reliability of BIA measurements rely on following procedures carefully [11,12]. It is recommended that participants are measured in a fasted state, exercise is avoided prior to measurements, and products containing caffeine are avoided prior to measurements. This is to reduce inaccuracies which occur as a result of shifts in body fluids. It was established in the previous paragraph that BIA uses resistance to estimate FFM, as it is related to TBW. Consuming large volumes of fluid will increase the TBW, lower resistance, and result in an increased FFM, as estimated by the BIA device. It has been established that this effect is transient and is influenced by the osmolality of the consumed fluid [13,14]. The effect is thought to be reduced over time as TBW is distributed across tissues.

Fluids containing caffeine such as coffee make for an interesting case, due to the specific absorption, distribution and excretion of water influenced by the caffeine. Caffeine is a methylxanthine found in over 60 plants, some of which humans consume for their psychoactive properties [15]. Common foods containing caffeine include coffee and tea, chocolate and cola drinks, with coffee, tea and caffeinated energy drinks being the largest contributors of caffeine in the UK diet [16]. It is thought that caffeine in high doses affects fluid shifts in the body by acting on the kidney, producing a diuretic effect [17]. However, the research on this is unclear, with evidence suggesting that moderate coffee consumption has the same hydrating effect as water in habituated, healthy males [18]. Water in coffee begins to be absorbed by the stomach and small intestine within approximately 10 to 15 minutes following consumption. Water and dissolved solutes, including caffeine and other methylxanthines continue to be absorbed in the small intestine, with peak blood concentrations observed at 45 minutes following consumption. Water from coffee is distributed to body tissues according to need, with more highly metabolically active tissues such as the muscles and organs taking on more water. The kidneys manage whole-body fluid homeostasis, which will excrete any excess fluid in healthy subjects. Caffeine competitively antagonizes adenosine receptors in nephrons, increasing glomerular filtration rate, and inhibiting adenosine mediated vasoconstriction. Caffeine inhibits sodium reabsorption, resulting in sodium and fluid loss from ECF and ICF, and a corresponding theoretical decrease in total body water. However, it is thought that this effect is small and that coffee has a net hydrating effect, particularly in habituated caffeine users.

In order to improve practices in measuring anthropometry, recommendations should be made to avoid unnecessary restrictions on research participants, patients and clients using BIA [19]. The aim of this study was to determine whether BIA measurements including body fat percentage, impedance, resistance and reactance are affected by coffee consumption at different caffeine concentrations.

## 2. Methods

### 2.1. Study design

**Figure 1.**
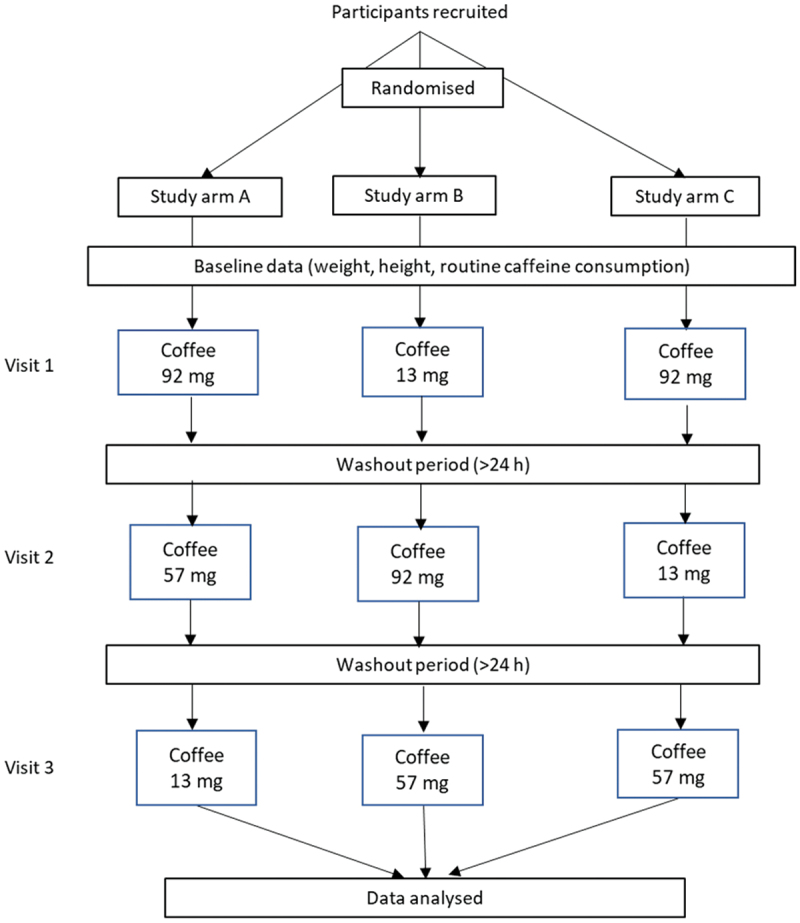
Summary of study design.

This research used a blinded, randomized, cross-over trial design, as shown in Figure 1.

### 2.2. Participants

Participants were drawn from students and staff at the researchers’ university. The study made use of a convenience sample design. The study included healthy males and females, between the ages of 18 and 59 years old. Participants with a body mass index (BMI) within the range of 18.5 to 29.9 kg/m^2^ were included. The study excluded participants who were pregnant, breastfeeding, had an implanted pacemaker or medical device, those using diuretics, steroid medications or supplements affecting water balance. Participants who had health conditions including heart disease, edema, kidney disease, chronic obstructive pulmonary disease (COPD), cancer or taking medication for any of these conditions were also excluded from the study.

### 2.3. Measurement techniques and procedures

#### 2.3.1. Caffeine content analysis from coffee samples

Nescafe Gold Blend and Nescafe Gold Blend Decaffeinated (Nestlé, Vevey, Switzerland) were purchased from a local supermarket. All other reagents and solvents used in these experiments were procured from Fisher Scientific, Loughborough, UK, unless stated otherwise. A sample of each coffee preparation was placed in a thimble and caffeine was extracted using 120 mL of ethanol under Soxhlet conditions for one hour. After, 13 g of magnesium oxide were added and the solution evaporated to dryness. The solid residue was then extracted with hot water and filtered. A 4 M HCl solution (2 mL) was then added to the cooled inorganic layer and extracted with dichloromethane (4 ×50 mL). The organic layer was dried with magnesium sulfate and evaporated to dryness. The caffeine residue was recrystalized and analyzed by nuclear magnetic resonance and quantified by high-performance liquid chromatography. ^1^H-NMR was recorded on a Bruker AV500 (Bruker, Massachusetts, United States) spectrometer operating at 500 MHz. Chemical (*δ*_H_) are quoted as parts per million downfield from 0. The multiplicity of a ^1^H-NMR signal is designated by one of the following abbreviations: s = singlet, d = doublet, *t* = triplet, q = quartet, quin = quintet, sept = septet, br. = broad and *m* = multiplet. Coupling constants (J) are expressed in Hertz.

Caffeine analysis was performed using a HPLC (Agilent Technologies 1200 series) equipped with an Agilent G1322A degasser, G1311A quaternary pump, G1329A autosample and G1316A thermostat column compartment (Agilent Technologies, Cheadle, UK). The analysis was done using a Phenomenex Luna Phenyl Hexyl column fitted with a guard column (Phenomenex, Cheshire, UK). The length, internal diameter and particle size were 250 mm, 4.6 mm and 5 μm, respectively. The mobile phase consisted of water:methanol (40:60). Prior to its use, the latter was degassed using an ultrasonicator (VWR International, Lutterworth, UK) to remove air bubbles. The flow rate of the mobile phase was 1 mL min^−1^, and the column temperature was set at 23°C. Data was acquired at a wavelength of 274 nm. A sample volume of 10 μL was injected for a total run time of 10 min. A known amount of caffeine (Sigma Aldrich, Dorset, UK) was dissolved in water (pH 7.3 ± 0.1), and a stock solution (1000 μg mL^−1^) was prepared. The stock solution was diluted to prepare various concentrations of caffeine. The caffeine peak was evident at 3.7 min. The calibration curve was constructed in the concentration range of 0.05–500 μg mL^−1^. A linear relationship was found between concentration and peak area with regression coefficient values (r^2^) of greater than 0.999. The LOQ and LOD values were 0.5 and 0.05 μg mL^−1^, respectively.

#### 2.3.2. Habitual caffeine use

Participants were asked to complete a validated questionnaire [20] to determine their habitual caffeine consumption. Consumption of caffeinated foods and beverages was calculated using the values per serving supplied by Buhler et al. [20].

#### 2.3.3. Anthropometric measurements and BIA

Weight was measured using techniques employed by the World Health Organization, using a Seca medical body composition analyzer (mBCA) 515 device (Seca GMBH & Co, Hamburg, Germany). Scales were calibrated prior to measurements. Measurements were taken in triplicate. Participants were asked to remove their shoes and were weighed in light clothing, in kilograms (kg), to the nearest 0.01 kg (10 g). Height was measured using a Leicester height measure (Marsden Weighing Machine Group Ltd, Henley-on-Thames, United Kingdom). Measurements were taken using techniques endorsed by the World Health Organization. Participants were asked to remove their shoes and headdress, and were measured in light clothing, in centimeters (cm), to the nearest 0.1 cm (1 mm). Participants were asked to stand with their back to the stadiometer, with the back of the head, shoulders, buttocks and heels against the stadiometer and head in the Frankfort plane position. The researcher took the measurement at eye level to prevent errors associated with parallax. Measurements were taken in triplicate. The same scale and stadiometer were used to measure all participants. Measurements were taken by fieldworkers trained in anthropometric measurements, with backgrounds in dietetics and sports physiology.

Body composition parameters were measured using the Seca mBCA 515 device. The device is a segmental, stand-on BIA system, with an integrated scale in the base platform, connected to a handrail that houses a display unit and the hand electrodes. Pairs of electrodes are positioned at each hand and foot, for a total of eight electrodes. During measurement, each forefoot is placed on an anterior electrode and each heel is placed on a smaller, posterior electrode. The electrodes that make contact with the hands are set in the handrail, which is positioned so that the arms are at a 30° angle to the body. The two electrodes contacting each hand are separated by a small plastic separator which is positioned between the middle and ring fingers. The electric current passes from one electrode per pair at each contact point with the limb, and the other electrode detects the change in voltage. This eight-electrode configuration enables segmental analysis of body composition. Measurements start automatically when the participant is correctly, making contact with each electrode. The device measures impedance using an electric current at 100 μA at 19 frequencies (1, 1.5, 2, 3, 5, 7.5, 10, 15, 20, 30, 50, 75, 100, 150, 200, 300, 500, 750 and 1000 kHz). Impedance, reactance, resistance and phase angle are reported by the device at 5 and 50 kHz. The device was calibrated prior to measurements. The platform and handrail were swabbed with an alcohol wipe prior to measuring each participant. Participants were seated between measurements, BIA measurements were taken per the manufacturer’s instructions and fat mass was estimated by the device using the manufacturer’s proprietary equations.

### 2.4. Treatment

Participants arrived at the laboratory in a 12-hour fasted state, at a consistent time in the morning to control for diurnal changes in body fluid, having been informed not to drink any alcohol the night prior to data collection. Participants were informed not to partake in any exercise apart from daily living activities for 24 hours prior to data collection.

Prior to starting the measurements and after the final BIA measurements, participants were asked to collect a urine sample. The urine sample was measured for osmolality using an Osmocheck PAL-OSMO pocket refractometer (Vitech Scientific Ltd). This device displays mOsm/kg H_2_O results and is calibrated to a range of 0–1500 mOsm/kg H_2_O. Participants emptied their bladders when taking the urine sample, and did not urinate again until after the final measurement, when a second urine sample was obtained and tested.

Participants consumed a cup of coffee prepared by the researchers. Participants received one of three treatments: 200 mL hot water (80°C) mixed with 5 g instant coffee (Nescafe Gold Blend) supplying ~92 mg caffeine; 200 mL hot water (80°C) mixed with 2.5 g instant coffee and 2.5 g decaffeinated coffee (Nescafe Gold Blend Decaffeinated) supplying ~46 mg caffeine; or 200 mL hot water (80°C) mixed with 5 g decaffeinated instant coffee containing ~13 mg caffeine. The coffee was not sweetened with sugar, artificial sweeteners or milk. The decaffeinated instant coffee masked the taste and aroma of the treatment and participant’s and researchers were blinded to which treatment each participant received. Each cup of coffee was presented in a coded polystyrene cup. The code on the coffee cup was recorded against the participants data. The participants were given 20 minutes to consume the coffee, and the time was recorded. A baseline BIA measurement was taken in triplicate, prior to consuming the coffee, with subsequent measurements taken after the coffee was finished, at 10-minute intervals (at 0 minutes, 10 minutes, 20 minutes, 30 minutes, 40 minutes and 50 minutes). Previous studies have established changes in BIA parameters occur within 15 to 30 minutes of ingesting fluids [19,21], and the study was limited to 50 minutes to avoid unnecessary discomfort to participants. After a washout period of at least 24 hours, the participants returned to the laboratory and the coffee consumption and BIA measurements were repeated. A final measurement took place after a second washout period of at least 24 hours.

### 2.5. Data capture

Participant characteristics were collected, including age and sex. Outcome measures included impedance (Z), resistance (R), reactance (Xc) and phase angle (ϕ) at 50 kHz and 5 kHz. Relative fat mass (%FM) was captured. Data collection techniques and tools, including the data capture sheet, were pilot tested prior to the full study. Data were captured onto a Microsoft Excel spreadsheet.

### 2.6. Statistical analysis

Statistical analysis was conducted using RStudio [22]. Continuous variables were reported as means with standard deviations. Categorical variables were reported as frequencies and relative frequencies. The device coefficient of variation (CV) was calculated as standard deviation/mean for impedance, resistance, reactance and phase angle at 50 kHz and 5 kHz from the measurements taken in triplicate prior to treatment being administered. Technical error of measurement (TEM) was calculated as TEM=$\sqrt{\Sigma d^{2}}\;/\;\text{\{2N\}}$, and used to ascertain the amount of the total standard deviation for the sample that is a result of measurement error, where d is the difference between measurements. Relative error was calculated as $\frac{\mathrm{TEM}}{\mathrm{mean}\;\mathrm{of}\;\mathrm{measurement}}$ and expressed as a percentage, where a lower value suggests higher precision. Coefficient of reliability (R) was calculated as *R* = 1 - (Total TEM^2^/SD^2^) and demonstrates the proportion of anthropometric variation between repeated measurements that is free of measurement error. Low variability between repeated measures is demonstrated by a value of R that is close to 1. These values were calculated to assess the level of precision and reliability of the SECA mBCA 515. Baseline differences between the sexes were assessed using independent t-tests. Changes from baseline and subsequent measures of BIA parameters were plotted and differences in impedance, resistance, reactance, phase angle, body fat percentage, fat-free mass percentage and urine osmolality were calculated using a three-way repeated measures ANOVA with *p* < 0.05 considered statistically significant. The normality assumption was tested using QQ plots. Participant sex, treatment and time were used as the predictors and interactions were tested for these variables. The Greenhouse-Gauser sphericity correction method was applied where appropriate. An additional model was developed, including habitual caffeine use was used as a covariate. The effect size was estimated using generalized eta squared (η^2^_G_). An a priori sample size was calculated using G*Power v3.1.9.7 [23]. The required sample size was 12 participants, given an effect size (f) of 0.4 at α = 0.05 and 80% power.

### 2.7. Ethics

Ethics approval for the study was obtained from the [removed for peer review] research ethics committee. This research was conducted in line with the principles of medical research using human participants set out in the Declaration of Helsinki [24]. Informed consent was obtained from participants before enrollment in the study. The nature and purpose of the study was explained to participants prior to enrollment.

## 3. Results

17 participants were recruited to the study and 13 participants completed the study. The average age of participants was 30.3 (±7.6) years. 30.8% (*n* = 4) of the sample were male and 69.2% (*n* = 9) were female. The average BMI was 23.15 (±1.87) kg/m^2^. The average caffeine intake was 183.9 (±103.1) mg per day. Mean caffeine per kilogram body weight was 2.85 (±1.57) mg/kg. Characteristics of the participants at baseline are presented in Table 1.

**Table 1.** Baseline characteristics of study participants (*n* = 13).

Characteristic	All (*n* = 13)	Male (*n* = 4)	Female (*n* = 9)	p
Age (years)	30.3 ± 7.6	37.7 ± 7.8	27 ± 4.6	.09
Weight (kg)	66.09 ± 8.39	71.49 ± 8.32	63.70 ± 8.22	.17
Height (m)	1.68 ± 0.07	1.75 ± 0.08	1.65 ± 0.05	.11
BMI (kg/m^2^)	23.15 ± 1.87	23.27 ± 1.93	23.09 ± 1.95	.88
Impedance (Z_50_) (Ω)	620.8 ± 80.8	530.1 ± 8.9	661.2 ± 61.9	< .01*
Impedance (Z_5_) (Ω)	702.8 ± 80.4	614.1 ± 6.0	742.3 ± 63.2	< .01*
Resistance (R_50_) (Ω)	617.8 ± 81.2	526.6 ± 9.26	658.3 ± 62.1	< .01*
Resistance (R_5_) (Ω)	701.8 ± 80.3	613.3 ± 6.0	741.1 ± 63.3	< .01*
Reactance (Xcr_50_) (Ω)	62.8 ± 4.9	60.6 ± 4.7	63.8 ± 4.9	.31
Reactance (Xcr_5_) (Ω)	30.2 ± 3.7	31.2 ± 3.7	29.8 ± 3.5	.55
Phase angle (ϕ_50_) (°)	5.9 ± 0.7	6.6 ± 0.6	5.6 ± 0.5	.03*
Phase angle (ϕ_5_) (°)	2.5 ± 0.4	2.9 ± 0.3	2.3 ± 0.3	.03*
Fat mass (%)	25.6 ± 7.1	14.9 ± 6.3	27.6 ± 4.7	.01*
Fat-free mass (%)	74.38 ± 7.1	85.1 ± 5.9	72.4 ± 4.7	.01*
Urine osmolality (mOsm/kgH_2_O)	606.2 ± 286.8	837.5 ± 160.9	503.3 ± 267.9	.03*
Caffeine intake (mg/day)	183.9 ± 103.1	260.1 ± 94.1	162.1 ± 100.9	.34

*Sex differences significant at *p* < 0.05 for Welch two-sample t-test.

Device technical error of measurement (TEM) was found to be 3.7 Ω for Z_50_ and 4.37 Ω for Z_5_, 3.85 Ω and 4.83 Ω for R_50_ and R_5_, 0.58 Ω and 0.59 Ω for Xc_50_ and Xc_5_ and be 0.05° and 0.03° for ϕ_50_ and ϕ_5_. Technical error of measurement was found to be very low for all measures, with relative error values below the 2% threshold for acceptable reliability (Table 2).

**Table 2.** Reliability measures of BIA outputs impedance, reactance, resistance and phase angle including CV, TEM, relative error and R.

BIA output	CV (%)		TEM	Relative error (%)	R
Z_50_	0.63		3.7 Ω	0.59	0.998
Z_5_	0.57		4.37 Ω	0.62	0.997
R_50_	0.7		3.85 Ω	0.62	0.997
R_5_	0.68		4.83 Ω	0.69	0.996
Xcr_50_	0.88		0.58 Ω	0.92	0.985
Xcr_5_	1.53		0.59 Ω	1.95	0.977
ϕ_50_	0.92		0.05°	0.87	0.995
ϕ_5_	0.65		0.03°	1.50	0.993
BIA parameter	Effect	d.f.	F	p	η^2^_G_
Z_50_	Sex	1, 11	18.37	0.001	0.599
	Treatment	1.75, 19.25	1.03	0.365	0.008
	Sex × treatment	1.75, 19.25	0.02	0.975	< 0.001
	Time	3.18, 35	4.30	0.01	0.003
	Sex × time	3.18, 35	3.44	0.025	0.002
	Treatment × time	4, 43.97	0.76	0.560	< 0.001
	Sex × treatment × time	4, 43.97	0.91	0.464	< 0.001
Z_5_	Sex	1, 11	16.93	0.002	0.571
	Treatment	1.71, 18.82	0.85	0.427	0.009
	Sex × treatment	1.71, 18.82	0.03	0.956	< 0.001
	Time	2.20, 24.19	10.16	< 0.001	0.005
	Sex × time	2.20, 24.19	2.89	0.071	0.001
	Treatment × time	3.77, 41.45	1.28	0.294	< 0.001
	Sex × treatment × time	3.77, 41.45	1.06	0.388	< 0.001
R_50_	Sex	1, 11	19.01	0.001	0.610
	Treatment	1.62, 17.86	0.82	0.433	0.006
	Sex × treatment	1.62, 17.86	0.02	0.956	< 0.001
	Time	2.38, 26.23	9.15	< 0.001	0.004
	Sex × time	2.38, 26.23	2.02	0.146	< 0.001
	Treatment × time	4.09, 44.99	1.11	0.364	< 0.001
	Sex × treatment × time	4.09, 44.99	0.63	0.648	< 0.001
R_5_	Sex	1, 11	17.25	0.002	0.576
	Treatment	1.72, 18.95	0.99	0.378	0.011
	Sex × treatment	1.72, 18.95	0.08	0.899	< 0.001
	Time	2.47, 27.18	11.61	< 0.001	0.005
	Sex × time	2.47, 27.18	2.07	0.137	< 0.001
	Treatment × time	4.77, 52.49	1.06	0.394	< 0.001
	Sex × treatment × time	4.77, 52.49	1.33	0.267	< 0.001
Xcr50	Sex	1, 11	0.05	0.822	0.004
	Treatment	1.84, 20.25	0.95	0.396	0.016
	Sex × treatment	1.84, 20.25	0.5	0.6	0.009
	Time	2, 22	N/a	n/a	< 0.001
	Sex × time	2, 22	n/a	n/a	< 0.001
	Treatment × time	4, 44	n/a	n/a	< 0.001
	Sex × treatment × time	4, 44	n/a	n/a	< 0.001
ϕ50	Sex	1, 10	10.08	0.01	0.474
	Treatment	2, 19.98	0.58	0.570	0.005
	Sex ×treatment	2, 19.98	0.69	0.513	0.006
	Time	3.34, 33.43	0.37	0.794	< 0.001
	Sex ×time	3.34, 33.43	1.90	0.144	0.001
	Treatment ×time	3.86, 38.58	0.98	0.428	0.001
	Sex ×treatment ×time	3.86, 38.58	0.94	0.449	0.001
ϕ5	Sex	1, 9	5.15	0.049	0.336
	Treatment	1.70, 15.34	0.47	0.602	0.005
	Sex ×treatment	1.70, 15.34	1.25	0.309	0.012
	Time	3.13, 28.14	1.99	0.135	0.002
	Sex ×time	3.13, 28.14	0.46	0.719	< 0.001
	Treatment ×time	10, 90	0.71	0.713	0.001
	Sex ×treatment ×time	10, 90	0.56	0.843	0.001
Fat mass (%)	Sex	1, 4	16.36	0.016	0.8
	Treatment	1.3, 5.22	1.05	0.377	0.001
	Sex ×treatment	1.3, 5.22	0.82	0.437	0.001
	Time	5, 20	5.43	0.003	0.005
	Sex ×time	5, 20	1.66	0.191	0.001
	Treatment ×time	10, 40	1.03	0.434	0.003
	Sex ×treatment ×time	10, 40	1.29	0.267	0.004
Fat free mass (%)	Sex	1, 4	16.36	0.016	0.8
	Treatment	1.3, 5.22	1.05	0.377	0.001
	Sex ×treatment	1.3, 5.22	0.82	0.437	0.001
	Time	5, 20	5.43	0.003	0.005
	Sex ×time	5, 20	1.66	0.191	0.001
	Treatment ×time	10, 40	1.03	0.434	0.003
	Sex ×treatment ×time	10, 40	1.29	0.267	0.004

### 3.1. Caffeine content analysis from coffee sample

Nuclear magnetic resonance analysis indicated the purity of caffeine from the coffee extractions. D_H_ (500 MHz, CDCl_3_) 7.49 (s, 1 H), 3.94 (s, 3 H), 3.51 (s, 3 H), 3.33 (s, 3 H). Quantification of caffeine from both coffee samples (*n* = 3) showed the presence of caffeine in the following ratios: Nescafe Gold Blend CAFFEINATED, 1 g of coffee comprised 18.46 ± 0.39 mg of caffeine and Nescafe Gold Blend DECAFFEINATED, 1 g of coffee comprised 2.61 ± 0.36 mg of caffeine. Values were in accordance with the supplier’s data.

Hence, for the coffee treatment group, participants received 92.3 mg caffeine, for the mixed treatment group, participants received 52.7 mg of caffeine and for the decaffeinated coffee group, participants received 13.05 mg of caffeine.

### 3.2. Coffee treatment

**Figure 2.**
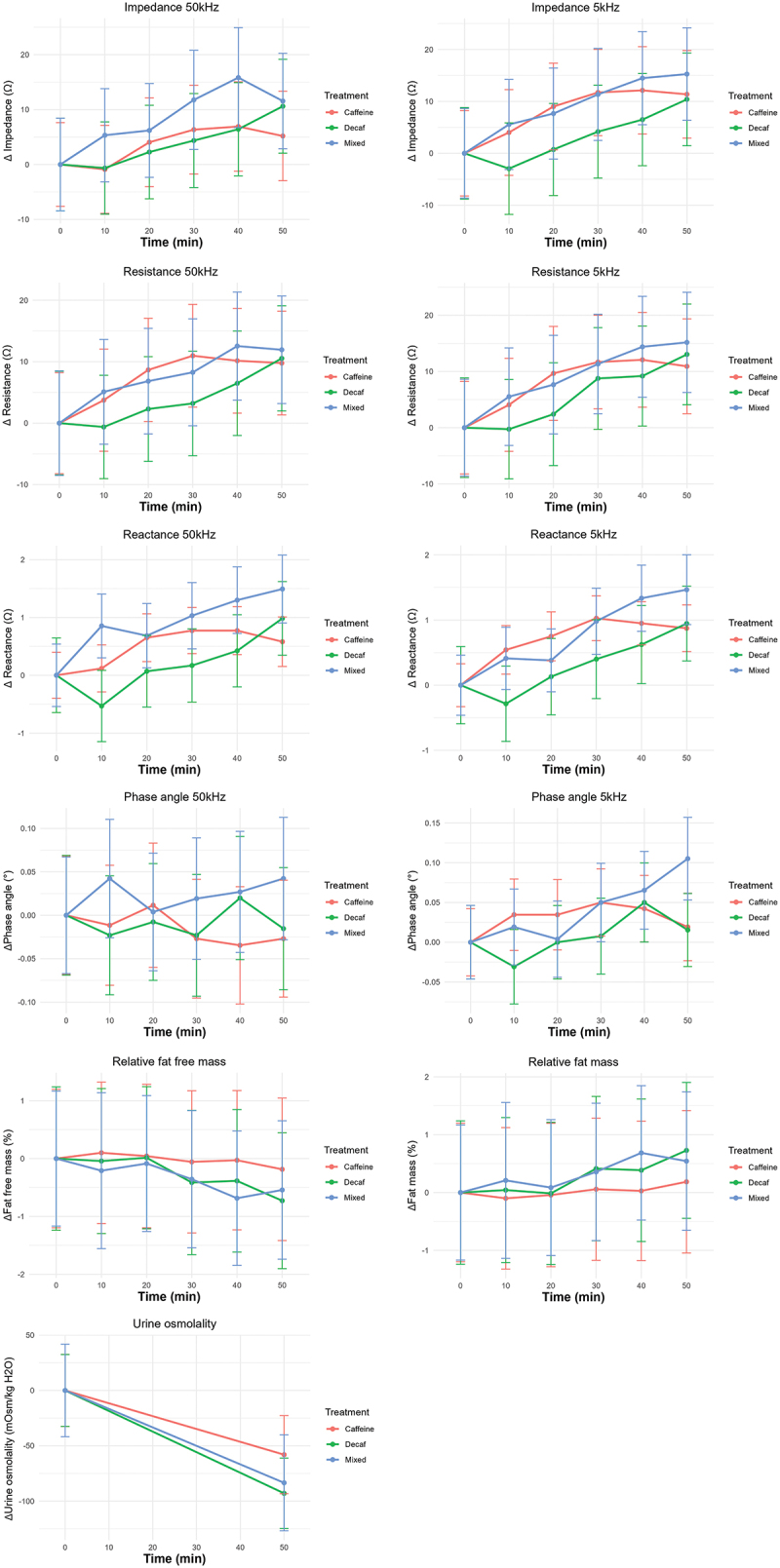
Changes in BIA parameters including impedance, reactance, resistance and phase angle at 50 kHz and 5 kHz and changes in fat free mass, fat mass and urine osmolality over 50 minutes, with different doses of caffeine in coffee; data is presented in ohms (Ω) for Z, R and Xc, and degrees (°) for ϕ (*n* = 13).

The treatment predictor (*p* > 0.05) and sex-time-treatment interaction for all outcomes was found to be non-significant (*p* > 0.05). The time predictor was statistically significant (*p* < 0.05) for impedance, resistance and reactance but not for phase angle ϕ50 (*p* = 0.731), ϕ5 (*p* = 0.059) or urine osmolality (*p* = 0.066). The sex predictor was statistically significant for Z_50_ (*p* = 0.001), Z_5_ (*p* = 0.002), R_50_ (*p* = 0.001), R_5_ (*p* = 0.002), ϕ_50_ (*p* = 0.01), ϕ_5_ (*p* = 0.049), fat mass (%) (*p* = 0.016) and fat free mass (%) (*p* = 0.016). The effect size for this predictor was η^2^_G_ < 0.336. A significant sex-time interaction was found for Z_50_ (*p* = 0.025) with a small effect size (η^2^_G_ < 0.01). When habitual caffeine intake was included as a covariate, none of the predictors were statistically significant for any of the BIA outputs (*p* > 0.05). General eta squared was calculated for each ANOVA test, and results for the treatment predictor, and time-treatment interaction were found to be small (η^2^_G_ < 0.01). Effect size for the time predictor was small (η^2^_G_ > 0.01). Results of the measurements are presented in Figure 2.

Urine osmolality was not different across treatment groups at baseline (F = 2.882, d.f. = 2;14, *p* = 0.7) nor at follow up (F = 0.39, d.f. = 2;12, *p* = 0.095) and treatment had no effect on urine osmolality (*p* = 0.377). The time (*p* = 0.003, η^2^_G_ = 0.005) and sex (*p* = 0.016, η^2^_G_ = 0.8) predictors were significantly associated with changes in urine osmolality but the interaction was non-significant (*p* = 0.191).

## 4. Discussion

This study investigated the influence of consuming caffeine in coffee, on the reliability of BIA parameters in adult subjects. It was found that the amount of caffeine in coffee did not affect BIA outputs, including impedance, resistance, reactance or phase angle. Effect sizes for these outputs were small, suggesting little practical significance of drinking coffee before taking BIA measurements. It was however, found that all parameters apart from phase angle increased over the course of the measurements, possibly as a result of the effect of water in the coffee. It was also found that the different concentrations of caffeine had no effect on urine osmolality at the end of 50 minutes. Previous studies and clinical recommendations have included avoidance of caffeine, or drinking coffee, in the hours preceding a BIA measurement as part of their protocols [11,12]. The results of this study suggest that such precautions are not necessary, and that consuming coffee prior to BIA measurements will not affect the reliability of measurements. Androutsos et al. [12] have already demonstrated that while food and fluid consumption results in statistically significant effects on BIA outputs, the effect sizes were small and therefore unlikely to be clinically significant.

This study used an octopolar Seca mBCA 515 device, which is a segmental BIA device that takes measurements with participants in a standing position. In the study by Androutsos et al. [12], a foot-to-foot BIA TANITA TBF-300 was used to measure impedance values. The Tanita TBF-300 model is a tetrapolar, foot-to-foot device that takes measurements in the standing position. This system shares the pressure plate system in common with the Seca mBCA 515 device, in which electrodes make contact with the soles of the feet and the palms of the hands and voltage is measured foot-to-foot and hand-to-hand. Matthews and Hosick [13] administered 466 mL of an isotonic drink to participants before measuring changes in plasma volume, serum sodium concentration and BIA impedance over 90 minutes. Plasma volume and serum sodium concentration increased over the experiment, but BIA impedance was unaffected [13]. Notably, this study used a single frequency, 50 kHz foot-to-foot BIA device and concluded that such a device would be unable to detect changes in body fluid. The alternative BIA device design is to use adhesive electrodes, with measurements typically taken in the supine position, and electrodes attached to the dorsal surface of the metacarpals and dorsal surface of the metatarsals or between the distal prominences of the radius and ulna and between the medial and lateral malleoli [25]. The results of this study, investigating the effects of drinking coffee with different concentrations of caffeine on BIA parameters, are similar to the findings of Mota et al. [19]. Recent research conducted by Mota et al. [19] has demonstrated that coffee consumption is associated with higher variance in measures associated with BIA, including body fat percentage, impedance, resistance and reactance up to 70 minutes after ingestion. Importantly, Mota et al. [19] used a Bodystat Quadscan 4000 device, which is a wrist to ankle device. Changes in BIA parameters including impedance, resistance and reactance changed after 30 minutes of ingesting coffee, while BIA outputs such as fat mass and fat free mass began to change after 45 minutes [19]. Mattioli [26] has questioned whether biological sex is an important determinant of fluid shifts associated with caffeine ingestion and subsequent measures from BIA. Bosy-Westphal et al. [10] have demonstrated that segmental devices such as the Seca mBCA 515 are more accurate than traditional wrist-to-ankle BIA. These researchers suggest that it is because the device is less sensitive to shifts in body water from the trunk to the limbs when compared to wrist-to-ankle devices, as the segmental BIA does not assume that the body is a single, perfect cylinder and therefore does not rely on the same assumption that water is not uniformly distributed across the body. Impedance, as measured by BIA, varies with conductor length and has an inverse relationship with cross-sectional area. Therefore, the trunk which contains a large proportion of body water, and arguably, the contents of the gastrointestinal tract, contributes less to whole-body impedance than the limbs, which are thinner. When comparing resistance at different points in the body, the ankles and wrists may account for greater than 50% of the total resistance measured, and the trunk accounts for only 40 Ω of resistance out of the ~ 500 Ω measured in a whole body measurement. However, Bosy-Westphal et al. [10] found no differences in accuracy when comparing standing and supine BIA measurements in segmental vs wrist to ankle devices. These researchers also challenged the assumption that BIA devices using pressure plate contact points are disadvantaged by the higher resistance in the bony ankles and wrists. In the study by Tinsley et al. [27], participants were required to stand for 65 minutes, resulting in increases In extracellular fluid detected in the legs for the time interaction only, and unaffected by treatment condition. Total body water decreased over the course of the experiment. Mota et al. [19] showed a similar decrease in TBW, ICF and ECF, except that participants were supine in between measurements.

The results of the tests of precision demonstrated that the SECA mBCA 515 produces results that have a high test-retest reliability. The technical error of measurement for impedance at 50 kHz was 3.7 Ω and 4.37 Ω at 5 kHz. During the experiment, changes to impedance (Z_50_) peaked at around 40 minutes, increasing by ~15 Ω from baseline in the mixed coffee group, and ~ 5 Ω in the decaf coffee group. Interestingly, the change to impedance continued to increase up to 50 minutes in the coffee group to 10 Ω. These peaks were outside of the range of technical error of measurement for the device, suggesting that the treatments raised the impedance, and that the differences were not as a result of natural variation in the reliability of the device. A similar pattern was observed for resistance at 50 kHz. Resistance peaked at 30 minutes for the decaf coffee group, at 40 minutes for the mixed group, and at 50 minutes for the coffee group. The peaks were at approximately 10 Ω higher than baseline for all groups, while the technical error of measurement was 3.85 Ω, suggesting that the changes were not due to natural variation in accuracy of the measurements. However, the increases in resistance for all treatments followed a similar pattern over the course of the experiment, and the ANOVA results suggest that the amount of caffeine in the coffee is not the factor that results in these changes. Furthermore, consuming fluid should theoretically reduce resistance, as conductivity increases and intracellular and extracellular fluids expand. Forejt et al. [28] demonstrated reduction in impedance, which as strongly associated with resistance, compared to baseline, 30 minutes after consumption of fluids when measured using BIA. However, this effect is perhaps limited due to the small contribution of the trunk to full-body resistance discussed above. Sagar et al. [29] showed gastric emptying of water and caffeine within approximately 30 minutes, with peak blood concentrations of caffeine appearing between 20 and 90 minutes following ingestion. Gastric emptying following fasting was fastest in the first 20 minutes following ingestion [29]. The peaks observed in impedance and resistance are possibly linked to the peak of appearance of caffeine in the blood and disappearance of water from the trunk and distribution to the muscles of the limbs. However, these results may also be as a result of normal diurnal changes observed in resistance or as a consequence of repeatedly standing and sitting between measurements. In either case, the effect of caffeine on fluid changes was not strong enough to overcome these more natural determinants of changes to resistance. Williamson et al. [21] tested the effects of 200 mg caffeine tablets versus a control on BIA outputs including relative fat mass and intracellular water. It was found that the treatment made no difference to the BIA outputs in habituated caffeine users [21]. These researchers used the Inbody 770 device for BIA measurements. The Inbody 770 takes 30 impedance measurements at 6 frequencies (1, 5, 50, 250, 500, 1000 kHz). This device is comparable to the Seca mBCA 515 as it also takes measurements in the standing position, using octopolar pressure contacts. Treatments and controls were consumed with 8 fluid ounces of water (~236 mL). The diuretic effect of caffeine is less pronounced in habitual coffee drinkers, and in the current study, the average daily caffeine consumption was the equivalent of two cups of coffee, per person per day, suggesting that the sample is habituated to caffeine. When habitual caffeine intake was included as a covariate in the present study, there were no significant differences detected across treatment groups. While some differences in urine osmolality were apparent across treatment groups in the present study, coffee treatment had no effect on urine osmolality compared to the control. In effect, both treatments and control hydrated participants. The fluid supplied by the water in coffee likely compensated for fluid losses resulting from any diuretic effect of caffeine in the coffee.

### 4.1. Strengths and limitations

A limitation of this study is that it used a convenience sample for data collection, possibly limiting generalizability of the results. A further limitation is the small sample size. Sex was neither modeled nor balanced in the current study. This study made use of a segmental octopolar, stand on device, and therefore the results of the study may not be applicable to devices that are tetrapolar, wrist-to-ankle, or require participants in a supine position for measurement.

Future research may improve knowledge in this area by comparing different types of BIA device, with different electrode configurations such as tetrapolar and octopolar devices. It would be valuable to compare results against a gold standard technique, such as air displacement plethysmography or DXA, to determine the effect of caffeine and fluid on accuracy and reliability. Future research might investigate male and female responses to caffeine in coffee [26], as there are sex-related differences in body compartments and water and electrolyte containing tissue. It may also be beneficial to test the effects of coffee consumption on BIA parameters in specific clinical groups where fluid balance is likely to be important and anthropometric data is clinically relevant.

## 5. Conclusion

Changes in impedance, resistance and reactance were detected over the course of the experiment, and these changes were greater than could be explained by the technical error of measurement of the Seca mBCA 515 device, however it was found that the amount of caffeine in coffee did not affect BIA outputs, including impedance, resistance, reactance or phase angle. Effect sizes for these outputs were small, suggesting little practical significance of drinking coffee before taking BIA measurements.
